# Personalized Exercise Training Modulates Red Blood Cell Rheology and Morphology in Long COVID

**DOI:** 10.3390/ijms27062671

**Published:** 2026-03-14

**Authors:** Anna-Lena Krüger, Frederieke Schmidt, Wilhelm Bloch, Björn Haiduk, Marijke Grau

**Affiliations:** 1 Molecular and Cellular Sports Medicine, Institute of Cardiovascular Research and Sports Medicine, German Sport University Cologne, 50933 Cologne, Germany; 2 S.P.O.R.T. Institut, Institute of Applied Sports Sciences, 51491 Overath, Germany

**Keywords:** long COVID, red blood cells, red blood cell deformability, red blood cell aggregation, exercise intervention, ferritin, fibrinogen, D-dimers, hematological parameters

## Abstract

Long COVID is associated with persistent fatigue, exercise intolerance, and microcirculatory dysfunction. Altered red blood cell (RBC) rheology, including impaired deformability and increased aggregation, may contribute to these symptoms, yet the effects of exercise interventions remain unclear. This longitudinal pilot study tested whether an individualized, symptom-responsive exercise program improves RBC rheology in Long COVID. A total of 170 (110 f/60 m) participants entered a five-phase training protocol; 15 completed all phases and formed a predefined finisher subgroup. RBC aggregation and deformability, hematological parameters, and coagulation- and iron-related markers were assessed across phases; RBC morphology was additionally analyzed in finishers at baseline and completion. In the total cohort, aggregation indices decreased across training phases, accompanied by prolonged aggregation half-time, while hematological, coagulation, and iron markers remained largely unchanged. The deformability changes were not uniform in the full cohort; however, finishers showed a deformability shift after completion. Importantly, morphologically abnormal RBC decreased in finishers, and these changes correlated with deformability, suggesting that improved rheology is linked to reduced RBC abnormalities. Prospectively, larger controlled studies are needed to confirm these results and to evaluate whether exercise-induced rheological improvements translate into functional and symptomatic benefits.

## 1. Introduction

Long COVID refers to a heterogeneous condition that is defined by the WHO as a post-COVID-19 condition with symptoms that begin or persist more than three months after a SARS-CoV-2 infection and last for at least two months and cannot be explained otherwise [1]. The most common symptoms include fatigue, exercise intolerance, cognitive impairment, and microcirculatory dysfunction [1,2,3]. In the context of Long COVID, exercise intolerance commonly manifests as an early onset of fatigue during physical activity, a reduced exercise capacity, and an exacerbation of symptoms after exertion, often described as post-exertional malaise (PEM) [4]. This impairment can be assessed objectively using cardiopulmonary exercise testing (CPET), which frequently shows a reduced peak oxygen uptake (VO_2peak_) and abnormal workload responses compared with individuals without persistent symptoms [5] and may reflect limitations in oxygen extraction [6] and autonomic regulation [7]. The symptoms can significantly impair quality of life and functional status in a considerable proportion of individuals recovering from COVID-19 [8,9,10]. A recent meta-analysis estimated the global pooled prevalence of Long COVID at approximately 36% among COVID-19 survivors [11]. The underlying pathophysiology is complex and multifactorial, involving immunological, inflammatory, neuroendocrine, and hemostatic alterations [12,13].

Altered hemorheology, particularly changes in red blood cell (RBC) deformability and aggregation, is an increasingly recognized aspect of Long COVID [14,15,16,17]. Both properties are essential for efficient oxygen transport and microvascular perfusion. Several studies have demonstrated reduced RBC deformability and increased aggregation even after mild SARS-CoV-2 infections, with effects often more pronounced in women [18]. These alterations are associated with elevated fibrinogen and D-dimer levels, suggesting persistent systemic inflammation and/or hypercoagulability in the post-acute phase [18]. However, a recent study reports persistently elevated RBC aggregation but increased RBC deformability in patients with Long COVID compared to non-Long COVID controls [15]. This alteration in deformability may result from changes in RBC morphology and/or the impairment of the cytoskeleton–membrane linkage, potentially leading to mechanically less stable cells, which may be more susceptible to irreversible deformation, membrane shedding, or premature clearance during repeated passage through the microvasculature [15,19,20]. Such instability may be driven by alterations in cytoskeletal proteins and membrane–cytoskeleton anchoring complexes, as well as changes in membrane lipid composition or oxidative stress-related damage [21,22]. Additionally, changes in hemoglobin and iron metabolism [23] have also been reported, all pointing toward the impaired microcirculation and oxygen delivery in Long COVID.

Notably, conventional laboratory markers such as hemoglobin, ferritin, and fibrinogen may show some alterations; however, their values often remain within normal ranges, suggesting that these changes can occur independently of standard clinical indicators.

Beyond mechanistic insights, potential interventions have come into focus. Evidence from exercise physiology suggests that regular physical activity can improve RBC properties and lower fibrinogen concentrations [24,25,26,27,28]. Exercise training significantly improves exercise capacity and reduces the severity of symptoms in individuals with Long COVID, with multiple studies demonstrating that structured exercise interventions lead to meaningful gains in physical function and symptom relief [29,30,31]. Targeting these interrelated processes through individualized exercise may offer multi-level benefits, including improvements in aerobic capacity and physical performance, reductions in fatigue and dyspnea, and enhancements in quality of life, while potentially also positively affecting the cellular mechanics, coagulation dynamics and microvascular function in individuals with Long COVID [29,30,31].

Therefore, the present study aimed to examine the effects of a data-driven and software-guided personalized exercise intervention, based on highly standardized protocols, on RBC rheological properties and related hematological and laboratory parameters in individuals with Long COVID. This program consists of a progressive increase in training intensity across five consecutive, structured phases. Thus, more specifically, the study pursued the following objectives:

1. To evaluate how RBC-related parameters and clinical markers change across distinct phases of the intervention program.
2. To analyze the subgroup of participants who completed the entire program (“finishers”), and to examine whether their outcomes differ from those of the total study cohort.

It is hypothesized that the personalized exercise intervention leads to improvements in RBC rheologic properties and favorable changes in fibrinogen, ferritin and/or D-dimer levels.

## 2. Results

### 2.1. Hematological RBC-Associated Parameters

#### 2.1.1. Presentation of Longitudinal Data of the Total Study Cohort

The outliers were identified and excluded from the dataset (Hb: *n* = 1; Hct: *n* = 1; RBC: *n* = 1; and MCH: *n* = 1). The data were normally distributed, and the differences between the phases were analyzed using REML. Statistically significant differences were observed only for MCHC (*p* = 0.0308), with pairwise differences between pre-phase and post-phase 2 (*p* = 0.0135), post-phase 3 (*p* = 0.0084), and post-phase 5 (*p* = 0.0458). No significant differences were detected for any other variables across the phases (Table 1).

#### 2.1.2. A Comparison of Initial Values Between the Total Study Cohort and Finishers, and a Comparison of Initial and Post-Phase 5 Data Among Finishers

The comparisons of initial values between the total cohort and the finisher subgroup, as well as comparisons between initial and post-phase 5 values within the finisher subgroup, are presented in Table 2. In the total cohort, implausible outliers were identified and excluded from the dataset (Hb: *n* = 1; Hct: *n* = 1; RBC: *n* = 1; and MCH: *n* = 1). No outliers were identified in the finisher subgroup. All of the datasets were normally distributed. The differences between initial values of the total cohort and the finisher subgroup were analyzed using unpaired *t*-tests, and a paired *t*-test was applied to assess the differences between initial and post-phase 5 values within the finisher subgroup. A significant difference in MCV was observed between initial values of the total cohort and the finisher subgroup (*p* = 0.0500). Within the finisher subgroup, RDW was significantly higher at post-phase 5 values compared with initial (*p* = 0.0475) values.

### 2.2. Rheological Parameters: Deformability and Aggregation Indices

#### 2.2.1. Changes in the Rheological Parameters of the Overall Study Group over the Course of the Study

**Figure 1:**
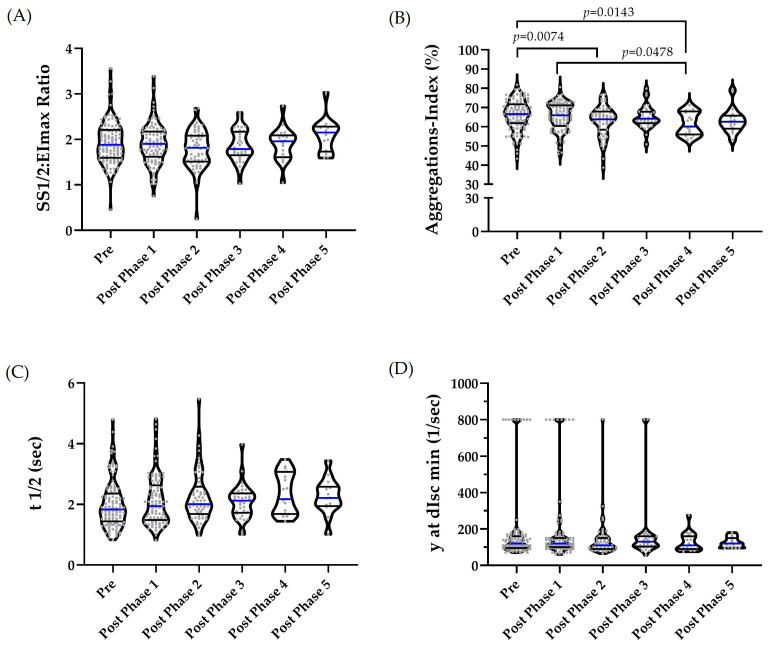
RBC deformability and aggregation indices of the total cohort during the intervention. After identifying and removing reasonable outliers, normality was assessed using the Shapiro–Wilk test. Where appropriate, log-transformation was applied to achieve a normal distribution of the data. A REML test was applied to identify differences during the intervention with a two-stage step-up method of BKY, as the post hoc test was applied where appropriate. (**A**) RBC deformability values did not differ between the phases (*p* = 0.0807). (**B**) The aggregation index significantly differed during the course of the intervention (*p* = 0.0347) with differences between pre/post 2 (*p* = 0.0074), pre/post 4 (*p* = 0.0143) and post1/post 4 (*p* = 0.0478). (**C**) Half-time of aggregation (t1/2) did not significantly differ between the phases (*p* = 0.2578). (**D**) The disaggregation–aggregation shear rate (γ at dIsc min) did not differ during the course of the study (*p* = 0.3319). The median is indicated by a blue line, while the percentiles are shown as black lines.

One outlier was identified in the pre-phase for RBC deformability; however, it was not excluded, as the subsequent value was comparable. REML revealed no significant overall effect (*p* = 0.0807) (Figure 1A). The RBC aggregation index showed no outliers. For t1/2, two outliers were identified in the pre-phase, three in post-phase 1, two that were each in post-phases 2 and 3, and one in post-phase 4. One value each from the pre-phase and one value each from post-phases 2 and 3 were excluded due to implausibility. The remaining values were retained, as they were deemed plausible based on intra-individual comparisons. For the aggregation–disaggregation shear rate γ at dIsc min, 14, 12, six and three outliers were identified in the pre-phase and in the post-phases 1–3, respectively. In the pre-phase, four outliers were removed; in post-phases 1 and 2, two outliers were removed each; and one outlier was removed from post-phase 3. The remaining values were retained following intra-individual comparisons. REML was used to assess the differences between phases. This analysis revealed a significant intervention effect for the aggregation index (*p* = 0.0347), with lower values observed at post-phase 2 (*p* = 0.0074) and post-phase 4 (*p* = 0.0143) compared with the pre-phase, and lower values at post-phase 4 compared with post-phase 1 (*p* = 0.0478) (Figure 1B). The values for t1/2 did not differ significantly among phases (*p* = 0.2578) (Figure 1C). The disaggregation–aggregation shear rate did not differ among phases (*p* = 0.3319) (Figure 1D).

#### 2.2.2. Comparison of Initial Values Between Total and Finisher Subgroup and the Initial vs. Post-Phase 5 Values Comparison of Finisher Subgroup

In the overall cohort, implausible values were identified (removed) from the dataset: *n* = 1 (1) SS1/2:EImax and *n* = 5 (5) for t1/2; *n* = 25 (8) for γ at dIsc min. No outliers were identified in the finisher group.

The data for SS1/2:EImax were normally distributed, and the differences between the initial total and the initial finisher were assessed using an unpaired *t*-test. The differences between initial and post-phase 5 values in the finisher subgroup were assessed using a paired *t*-test. The Mann–Whitney test was applied to assess the group differences between the total and initial finisher values, and a paired *t*-test was used for the differences between initial and post-phase 5 values in finishers. For γ at dIsc min, the differences between the initial values of the total cohort and the finisher subgroup were assessed using the Mann–Whitney test, and the differences between initial and post-phase 5 values in finishers were evaluated using the Wilcoxon matched-pairs signed-rank test.

**Figure 2:**
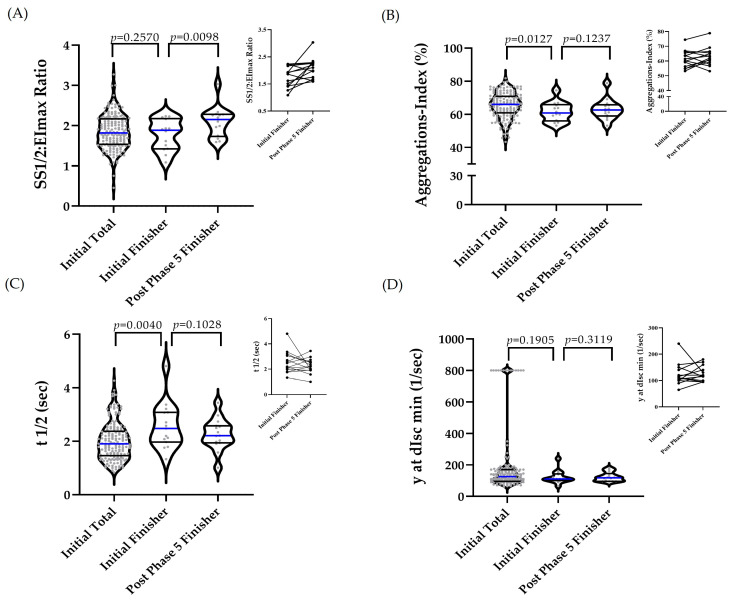
RBC deformability and aggregation parameters at the baseline and after the completion of the intervention. Parameters were compared between the initial total cohort and the initial finisher subgroup and within the finisher subgroup between the initial and post-phase 5 values. Although three groups are displayed for visualization, no overall three-group comparison was performed; only the specified pairwise comparisons were tested. The outliers were identified and removed where appropriate. For RBC deformability, the differences between the initial total and the initial finishers were analyzed using an unpaired *t*-test, and differences between the initial finisher and post-phase 5 finisher were analyzed using a paired *t*-test. For the aggregation index and aggregation half-time (t1/2), group differences between initial total and initial finishers were tested using the Mann–Whitney test, while the initial finisher vs. post-phase 5 finisher was analyzed using a paired *t*-test. The differences in the disaggregation–aggregation shear rate (γ at dIsc min) between the initial total and initial finisher were assessed using the Mann–Whitney test, and the Wilcoxon matched-pairs signed-rank was used to test for differences between the initial finisher vs. post-phase 5 finisher. The inset panels illustrate the paired individual trajectories of finishers between initial and post-phase 5 values, highlighting within-subject changes. (**A**) RBC deformability: Values were comparable between the total cohort and finishers (*p* = 0.2570) but differed significantly between the initial finisher and post-phase 5 finisher (*p* = 0.0098). (**B**) Aggregation index: Initial values were lower in finishers compared with the total cohort (*p* = 0.0127), with no significant change between the initial finisher and post-phase 5 finisher (*p* = 0.1237). (**C**) Aggregation half-time (t1/2): Values were higher in finishers compared with the total cohort at initial (*p* = 0.0040), with no significant change between the initial finisher and post-phase 5 finisher (*p* = 0.1028). (**D**) The disaggregation–aggregation shear rate (γ at dIsc min): No significant differences were observed between groups (*p* = 0.1905) or between the initial finisher and post-phase 5 finisher (*p* = 0.3119). The median is indicated by a blue line, while the percentiles are shown as black lines.

RBC deformability was comparable between the total cohort and the finisher subgroup at the start of the intervention (*p* = 0.257). In finishers, values increased, representing lower deformability at post-phase 5 (*p* = 0.0098) (Figure 2A). The aggregation index was significantly lower in finishers on initial assessment compared with the total cohort (*p* = 0.0127), with no significant change from initial to post-phase 5 values in finishers (*p* = 0.1237) (Figure 2B). The aggregation half-time (t1/2) was significantly higher in finishers on initial assessment compared with the total cohort (*p* = 0.0040) but did not significantly change from initial to post-phase 5 values in finishers (*p* = 0.1028) (Figure 2C). The aggregation–disaggregation shear rate γ at dIsc min (1/s) showed no significant differences between groups or time points (Figure 2D).

In the total cohort, the time since symptom onset showed a weak positive correlation with the initial RBC aggregation index (r = 0.16, *p* = 0.044) and a moderate positive correlation with the initial SS1/2:EImax (r = 0.31, *p* < 0.001), while no significant association was observed for the aggregation half-time (r = −0.14, *p* = 0.63). Overall, the correlation coefficients were small to moderate, indicating that disease duration explains only a limited fraction of the variability in the baseline rheological parameters and is therefore of modest clinical relevance. Within the finisher subgroup, no significant associations were observed between the time since symptom onset and the exercise-induced changes in the RBC aggregation index (r = −0.16, *p* = 0.56) or SS1/2:EImax (r = −0.39, *p* = 0.15), suggesting that the rheological changes are compatible with a training-related effect and argue against disease duration as a major driver of the observed rheological adaptations.

#### 2.2.3. Alterations in RBC Morphology and Their Association with Deformability in the Finisher Subgroup

The outlier analysis revealed no extreme values. Normality testing using the Shapiro–Wilk test indicated non-normal distributions; consequently, initial and post-phase 5 values were compared using the Wilcoxon signed-rank test. The proportion of morphologically altered RBC decreased from 12.3 ± 10.7% at the beginning of the program to 6.97 ± 6.31% post-phase 5 (*p* = 0.0105), representing an absolute reduction of 5.3 percentage points. Changes in the proportion of morphologically altered RBC were strongly correlated with changes in SS1/2:EImax from initial to post-phase 5 (Spearman r = −0.6003), indicating that decreases in morphologically altered RBC were associated with increases in SS1/2:EImax.

### 2.3. Coagulation Marker (D-Dimer and Fibrinogen) and Marker of Iron Metabolism (Ferritin)

#### 2.3.1. Changes in D-Dimer, Fibrinogen and Ferritin over the Course of the Study in the Overall Study Group

**Figure 3:**
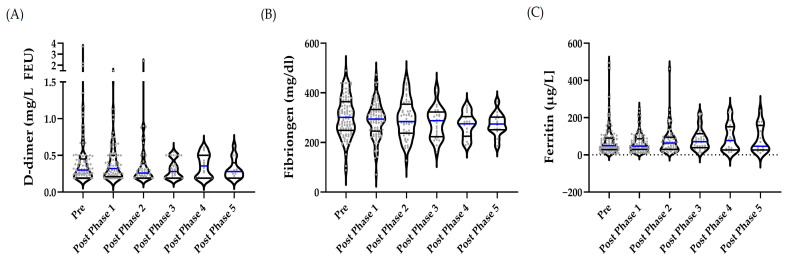
The coagulation and iron metabolism marker of the total study cohort during the intervention. No outliers were removed. Phase differences were assessed using REML. (**A**) D-dimer (*p* = 0.6154), (**B**) fibrinogen (*p* = 0.1887), and (**C**) ferritin (*p* = 0.2265) concentrations did not differ significantly between time points. The median is indicated by a blue line, while the percentiles are shown as black lines.

In the overall cohort, outliers were identified as follows: *n* = 13 in pre, *n* = 9 in post-phase 1, and *n* = 11 in post-phase 2. No outliers were detected for fibrinogen, while outliers for ferritin were *n* = 6, *n* = 6, and *n* = 3 in pre and post-phase 1, and post-phase 2, respectively. Outliers were retained because the values were considered reasonable based on intra-individual comparisons or because of the wide normal range reported for these parameters. REML revealed that concentrations of D-dimer, fibrinogen, and ferritin did not differ significantly between time points (*p* = 0.6154; *p* = 0.1887; *p* = 0.2265, respectively) (Figure 3A–C).

The correlation analysis revealed a strong positive correlation between fibrinogen and the aggregation index (r = 0.6556), a strong negative correlation between fibrinogen and the half-time of aggregation (r = −0.6620), and a moderate to positive correlation between fibrinogen and the disaggregation–aggregation shear rate (r = 0.5107). A table showing the correlations (Supplementary Table S1) can be found in the Supplementary Materials.

#### 2.3.2. A Comparison of Initial Values Between the Total and Finisher Subgroup and a Comparison of the Initial vs. Post-Phase 5 Finisher Subgroup

In the overall cohort, *n* = 24 and *n* = 11 outliers were identified for D-dimer and ferritin, respectively, but were not removed as the values were either plausible or expected given the wide reference range. The data of finishers showed no outliers. The differences between the initial total and initial finisher were assessed using the Mann–Whitney test for D-dimer and ferritin, and an unpaired *t*-test for fibrinogen. The differences between the initial finisher and post-phase 5 values were analyzed using the Wilcoxon matched-pairs signed-rank test.

**Figure 4:**
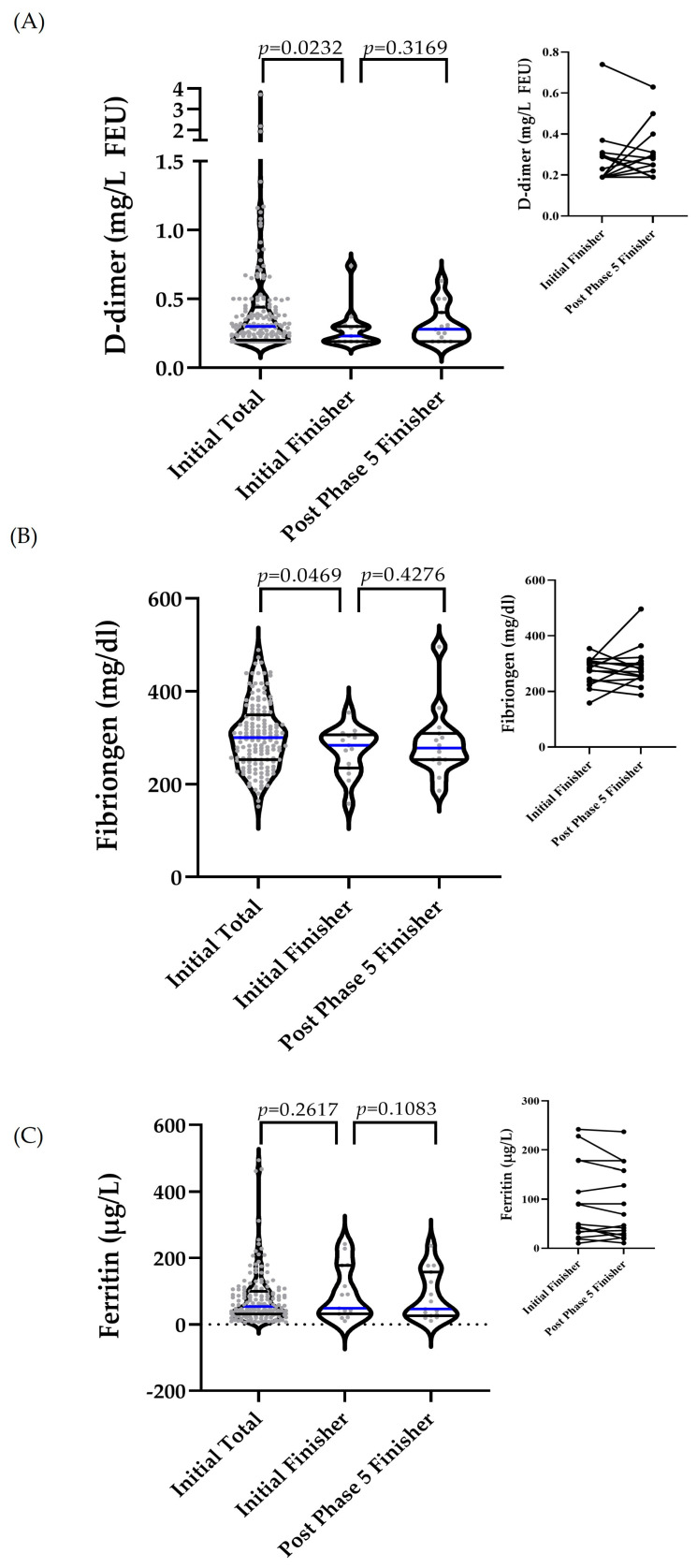
The coagulation and iron metabolism markers at the baseline and after the completion of the intervention. Markers were compared between the initial total cohort and initial finishers, and within the finisher subgroup between initial and post-phase 5 values. Although three groups are displayed for visualization, no overall three-group comparison was performed; only the specified pairwise comparisons were tested. The differences between the initial total and the initial finisher were analyzed using the Mann–Whitney test (D-dimer, ferritin) or an unpaired *t*-test (fibrinogen). The differences between the initial finisher and post-phase 5 were analyzed using the Wilcoxon matched-pairs signed-rank test. The inset panels illustrate the paired individual trajectories of finishers between initial and post-phase 5, highlighting within-subject changes. (**A**) D-dimer: Initial values were significantly higher in the total cohort compared with finishers (*p* = 0.0232), with no significant change between the initial finisher and post-phase 5 (*p* = 0.3169). (**B**) Fibrinogen: Initial values were significantly higher in the total cohort compared with finishers (*p* = 0.0469), with no significant change between the initial finisher and post-phase 5 (*p* = 0.4276). (**C**) Ferritin: Initial concentrations were comparable between the total cohort and finishers (*p* = 0.261), with no significant change between the initial finisher and post-phase 5 (*p* = 0.1083). All values are mean ± SD. FEU = fibrinogen equivalent units. The median is indicated by a blue line, while the percentiles are shown as black lines.

Initial D-dimer values were significantly lower in finishers compared with the total study group (*p* = 0.0232), while no significant change was observed between the initial and post-phase 5 values of finishers (*p* = 0.3169) (Figure 4A). Similar results were observed for fibrinogen, with lower initial values in finishers compared with the total study group (*p* = 0.0469) and no significant change from the initial and post-phase 5 in finishers (*p* = 0.4276) (Figure 4B). Initial ferritin levels were comparable between total and finishers (*p* = 0.2617) and remained unchanged at post-phase 5 in finishers (*p* = 0.1083) (Figure 4C).

## 3. Discussion

This study investigated whether a software-guided, symptom-responsive personalized exercise intervention (TRIBAL) program modulates red blood cell (RBC) rheology in individuals with Long COVID. By combining longitudinal analyses of the total cohort with the paired analyses of program finishers, the study examined exercise-associated adaptations in RBC deformability, aggregation, and related hematological, coagulation, and iron metabolism markers under real-world rehabilitation conditions.

The main findings indicate that personalized, symptom-limited exercise modulated RBC aggregation dynamics at the cohort level, while hematological parameters, coagulation markers, and ferritin concentrations remained stable. Importantly, finishers exhibited distinct baseline rheological and coagulation profiles and showed significant adaptations in RBC deformability and morphology during the intervention. Together, these findings highlight the heterogeneity of Long COVID and the potential of individualized exercise training to modulate hemorheology without adverse coagulation effects.

### 3.1. Hematological Parameters and Iron Metabolism

Consistent with previous findings, hematological parameters and ferritin concentrations were within reference ranges, indicating no overt hematological pathology despite persistent Long COVID symptoms [14,15,18].

In line with previous exercise intervention and controlled training studies, no systematic or sustained changes in red blood cell count, hemoglobin, hematocrit, or red cell indices were observed following moderate or individualized exercise [32,33,34,35]. Although MCHC fluctuated slightly, the magnitude was small and unlikely to reflect a training-induced effect [33,36]. Therefore, sampling-related factors appear to be a more plausible explanation, despite standardized blood collection procedures. Similarly, within the finisher subgroup, hematological parameters and ferritin concentrations remained stable from initial assessment to post-phase 5, except for an increase in RDW from initial to post-phase 5. The isolated increase in RDW that was observed in finishers may reflect an exercise-related increase in erythrocyte turnover [37] and population heterogeneity, rather than an adverse hematological effect, particularly given the stability of hemoglobin and ferritin levels. These findings suggest that personalized exercise intervention neither adversely affected erythropoiesis nor disrupted iron homeostasis.

### 3.2. RBC Rheology and Associated Coagulation Markers

Altered RBC rheology is increasingly recognized as a persistent feature of Long COVID, with previous studies reporting elevated RBC aggregation, altered deformability, and morphological abnormalities long after acute infection [15,18]. The initial aggregation index values observed in the present cohort were comparable to those previously reported in Long COVID populations and exceeded values typically reported for healthy controls [15], supporting the presence of sustained hemorheological alterations.

The baseline aggregation-related parameters showed only weak associations with the time since symptom onset, indicating no evidence of spontaneous normalization. Within the finisher subgroup, the changes in rheological parameters were not associated with the disease duration, suggesting that the observed improvements occurred independently of the time since symptom onset. Instead, longitudinal analyses demonstrated systematic changes in rheological parameters over the course of the intervention, supporting a temporal association with the individualized exercise program rather than a spontaneous recovery. In this context, it should be noted that although the intervention involved low absolute workloads, training was performed at individual symptom-limited capacity, resulting in a substantial relative physiological stimulus. Similar exercise studies have shown that even moderate or individualized training protocols can elicit measurable changes in RBC rheological behavior when the relative physiological demand is sufficient [38]. Over the course of the intervention, RBC aggregation dynamics were significantly modulated at the cohort level, as reflected by reductions in the aggregation index. These changes are in line with the recent literature [24,38] and occurred in the absence of significant alterations in fibrinogen or D-dimer concentrations. Nevertheless, strong correlations between fibrinogen and aggregation parameters underscore the relevance of plasma protein-RBC interactions for aggregation behavior, even within normal concentration ranges. Fibrinogen has been identified as one of the primary plasma proteins influencing RBC aggregation, with significant associations between fibrinogen concentration and key aggregation metrics [39]. The experimental studies further demonstrate that fibrinogen promotes the formation and stabilization of RBC clusters even at physiological concentrations [40]. Mechanistically, specific interactions between fibrinogen macromolecules and the RBC membrane appear to contribute to aggregation tendencies, supporting the biological relevance of plasma protein–cell surface interactions [41].

The analysis of the finisher subgroup revealed a distinct hemorheological profile. Finishers exhibited significantly lower aggregation index values and longer aggregation half-times at initial compared with the total cohort, alongside lower fibrinogen and D-dimer concentrations. Previous studies have shown that lower RBC aggregation and prolonged aggregation kinetics are associated with more favorable hemorheological conditions and reduced plasma protein-mediated cell–cell interactions [40]. Lower fibrinogen and D-dimer concentrations have been linked to a better functional capacity and a reduced thrombo-inflammatory burden, particularly in post-COVID and chronic disease populations [42]. Finishers also exhibited a more favorable functional status at the baseline, as reflected by lower post-COVID-19 functional status scale (PCFS) scores compared with the total cohort, while post-COVID syndrome score (PCS) values were numerically but not statistically lower. Together, these findings are consistent with the evidence indicating that individuals with preserved functional capacity tend to display more favorable baseline rheological and coagulation profiles, which may support sustained participation in exercise-based interventions [38]. This selective retention highlights the pronounced heterogeneity of Long COVID and emphasizes the importance of stratified analyses and responder characterization in future intervention trials.

RBC deformability followed a different pattern. While no significant change was observed in the total cohort, finishers showed a significant reduction in deformability from initial assessment to post-phase 5, indicating a shift in RBC mechanical properties. One possible explanation is exercise-associated adaptations in erythropoiesis and RBC maturation, which have been reported in endurance training studies [43,44,45]. Such adaptations may contribute to the structural stabilization of RBC and to the normalization of mechanical properties following SARS-CoV-2 infection [15,19,46]. Within this context, the observed reduction in deformability may reflect a normalization of RBC mechanical properties rather than a detrimental adaptation. This interpretation is supported by the significant reduction in the morphologically altered RBC in finishers and the strong correlation between improvements in RBC morphology and changes in deformability. Together, these findings suggest that personalized exercise training may promote the structural and functional stabilization of RBC in Long COVID.

It is important to note that excessive RBC aggregation and impaired deformability represent pathological alterations that may limit capillary perfusion and oxygen delivery [47,48]. In contrast, the exercise-associated reductions in aggregation [38], which are in line with previous findings, together with the normalization of deformability observed in the present study, may facilitate improved microvascular flow and oxygen transport, potentially contributing to functional recovery. Although these mechanistic interpretations remain to be conclusively demonstrated, they provide a biologically plausible framework linking exercise-induced modulation of erythropoiesis, RBC maturation, and membrane stability to improvements in the blood rheology that have been observed in Long COVID.

### 3.3. Methodological Considerations and Limitations

Certain methodological considerations should be taken into account when interpreting the present findings. While the exploratory single-arm design and the lack of a control group limit causal inference, the study aimed to assess changes in impaired red blood cell rheology during individualized exercise-based rehabilitation under real-world conditions. The participants entered the program with markedly reduced physical capacity, in some cases initiating training at workloads as low as 9 W. Under these conditions, the implementation of a comparable protocol in healthy individuals would be neither feasible nor clinically meaningful, limiting the applicability of traditional healthy control designs.

Attrition across training phases resulted in a small finisher subgroup; however, this was not driven by selection bias but rather reflected individual disease trajectories and symptom tolerance, underscoring the pronounced heterogeneity of Long COVID. Nevertheless, the longitudinal within-subject design allowed each participant to serve as their own control, reducing inter-individual variability in a highly heterogeneous Long COVID population. Standardized blood sampling procedures and consistent rheological methodologies further strengthen internal validity.

The relatively narrow age range of the cohort (mean age approximately 50 years) reflects the demographic characteristics of individuals seeking structured rehabilitation for persistent Long COVID symptoms. As older age has been associated with increased prevalence and persistence of Long COVID symptoms in large observational studies and meta-analyses, the present findings may not be generalizable to substantially younger or older populations [49]. Future studies should explicitly address age-dependent hemorheological responses to exercise in Long COVID.

### 3.4. Physiological and Clinical Implications

The observed modulation of RBC aggregation dynamics and favorable changes in deformability and RBC morphology suggest improved hemorheological efficiency and microvascular passage. Given that impaired blood fluidity and microcirculatory dysfunction have been implicated in Long COVID-related fatigue and exercise intolerance [50,51], these adaptations may represent a relevant physiological mechanism supporting rehabilitation outcomes.

While the magnitude of exercise-induced hemorheological adaptation varies widely across studies [24], the present findings support the concept that low-intensity, symptom-guided exercise can influence RBC rheology without provoking adverse coagulation responses in individuals with Long COVID. Personalized, software-guided training approaches may therefore offer a clinically feasible strategy to address persistent physiological impairments while minimizing the risk of symptom exacerbation.

## 4. Materials and Methods

### 4.1. Study Design

This is a prospective, single-arm, longitudinal observational study that was conducted between 1 November 2022 and 17 December 2025 in individuals with Long COVID. The investigation was embedded in a routine outpatient rehabilitation program and was not designed as an isolated experimental study but rather reflects the real-world therapeutic setting of an individualized exercise intervention.

All participants who enrolled in the rehabilitation program were eligible for inclusion, and no randomization, parallel control group, or predefined intervention duration were applied. Repeated measurements were taken at baseline (“initial”) and after each of the five training phases (post-phase 1–5) under standardized conditions.

### 4.2. Study Cohort/Participants

The participants were individuals who were currently experiencing Long COVID symptoms and required a formal medical diagnosis of their post-COVID condition (ICD-10: U09.9), issued by a primary care physician, at the time of the study inclusion. The exclusion criteria included acute infections, contraindications to exercise, severe anemia, and high-risk anticoagulation. The study followed an exploratory pilot/feasibility design targeting a medium effect size (Cohen’s d ≈ 0.5) to inform future larger trials. The study protocols were approved by the Ethics Committee of the German Sport University Cologne (171/2022) and were conducted in accordance with the principles of the Declaration of Helsinki. All of the participants received verbal and written information about the study and provided their written informed consent prior to participation. Table 3 summarizes the general information of the total study cohort (Total) and the subgroup called “Finisher”. “Finishers” are understood to be the participants who successfully completed the intervention program described below by the end of the observation period. In addition, the time interval between the onset of Long COVID symptoms and the initiation of the TRIBAL program was retrospectively assessed. In the overall cohort, the mean duration was 90.4 ± 53.3 weeks, while finishers initiated the program after 68.3 ± 35.1 weeks. Baseline symptom severity was assessed using the post-COVID Functional Status scale (PCFS) and the post-COVID syndrome score (PCS, according to Bahmer et al. [52]). The group differences between the total cohort and the finisher subgroup were evaluated using unpaired statistical tests.

### 4.3. Description of Co-Morbidities and Medication

Of the total of 170 participants, 48 (21 m/27 f) (28.2%) reported no pre-existing conditions, including two finishers. Orthopedic disorders were reported by 38 (15 m/23 f) participants (22.4%) in the overall cohort and by four finishers (28.6%). Cardiovascular-related disorders were documented in 29 (12 m/17 f) participants (17.1%) overall and in one finisher (7.1%). Thyroid disorders were reported by 32 (2 m/30 f) participants (18.8%) and by three finishers (21.4%). Respiratory diseases, such as asthma or COPD, were noted in 26 (7 m/19 f) participants (15.3%) overall and in two finishers (14.3%). Diabetes was reported by eight (4 m/4 f) participants (4.7%) in the overall cohort and by one finisher (7.1%).

In terms of medication use, 22 participants (10 m/12 f) (12.9%) in the overall cohort and two finishers (14.3%) reported not taking any medication. Ibuprofen, Acetylsalicylic Acid, or other painkillers were used by 80 (22 m/58 f) participants (47.1%) overall and by five finishers (37.7%). Blood thinners were taken by seven (1 m/6 f) participants (4.1%) overall, but by none of the finishers. Blood pressure medication was reported by 48 (19 m/29 f) participants (28.2%) overall and by two finishers (14.3%). Thyroid medication was used by 41 (5 m/36 f) participants (24.1%) overall and by three finishers (21.4%). Hormone use was documented in nine (1 m/8 f) participants (5.3%) overall and in one finisher (7.1%). Among the 110 women, four (3.6%) reported using contraceptives; none of the finishers reported contraceptive use.

Co-morbidities and medication use were documented to characterize the study cohort. These factors were not used as exclusion criteria, as the study employed a longitudinal within-subject design, with each participant serving as their own control across repeated measurements. Consequently, stable pre-existing conditions and long-term medication use were not expected to substantially influence relative changes in red blood cell rheological properties over the course of the intervention. Moreover, while conditions such as diabetes [53] or thyroid disorders [54] may affect hemorheological parameters, their prevalence was low in the cohort and therefore unlikely to have substantially influenced the overall results. No acute medication changes were reported during the study period.

### 4.4. Individual Training Program and Measurement Points

The participants engaged in an individualized and symptom-responsive exercise program following the TRIBAL protocol (tailored rehabilitation intervention based on activity for Long COVID). The intervention was based on core principles of pacing, post-exertional symptom monitoring, and progressive, low-threshold training stimuli. Training sessions were performed two to three times per week, with progression through five adaptive phases depending on individual tolerance and symptom development. A detailed description of the TRIBAL protocol has been previously reported [29].

**Figure 5:**
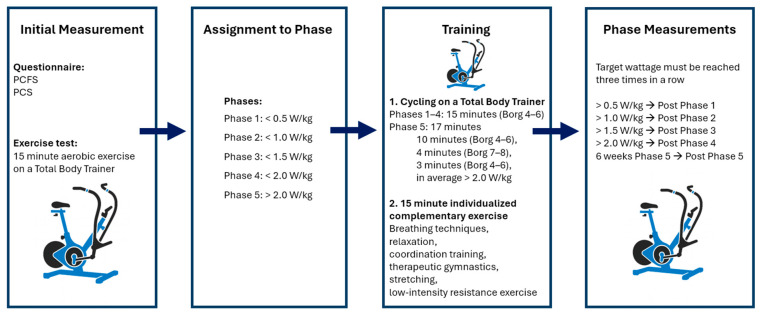
An overview of the measurement and training procedure. An initial assessment was conducted, consisting of the PCFS questionnaire and a 15 min aerobic exercise test on a Total Body Trainer, to assign participants to one of five phases based on wattage per kilogram. Training sessions follow the same structure and include cycling on the Total Body Trainer. Phases 1–4 involved 15 min of cycling at Borg CR10 levels 4–6, while phase 5 consisted of a 17 min protocol with alternating Borg CR10 levels (4–6 and 7–8), averaging >2.0 W/kg. The second part of the training session involved tailored content (e.g., stretching, breathing, and strength exercise). Once a participant reached the target wattage of the next phase on three consecutive training sessions, a phase measurement was performed. PCFS = post-COVID-19 functional status scale; PCS = post-COVID syndrome score.

As shown in Figure 5, at the start of the intervention, participants underwent a comprehensive assessment including the PCFS [55] and the PCS [52]. Additionally, participants completed a 15 min cycling test on a Total Body Trainer (Keiser, Fresno, CA, USA), which is a combined arm–leg ergometer enabling low-impact whole-body exercise. This cycling test was performed at 50 to 60 RPM and a physical exertion level between four and six on a modified Borg scale. The modified Borg-CR scale (Category Ratio Scale, CR10-Scale), ranging from 0 to 10, was chosen to simplify reporting and improve comprehensibility for patients with Long COVID-related fatigue and cognitive symptoms. It was used to determine the perceived exertion during and after cycling. This means that no wattage was specified and that cycling is adjusted (pacing) to avoid negative effects. The achieved wattage during this baseline test determined the initial training phase. The participants capable of maintaining a workload of <0.5/1/1.5 W/kg body weight on the whole-body ergometer were assigned to phase 1, 2 or 3, respectively.

For participants starting in phase 1, the baseline assessment was defined as “Pre”, and subsequent measurement points were labeled post-phase 1–5, according to the phase completed. Not all participants necessarily began in phase 1. The exercise intensity of the training sessions was prescribed based on initial performance diagnostics, and progression within the program was strictly criteria-based (subjective exertion, fatigue levels, and symptom feedback) and data-guided. This ensured that the training stimulus was only increased when the body had established sustainable tolerance, thus avoiding PEM [4].

Outcome measurements were repeated after the completion of each phase (post-phase 1–5). Progression to the next phase required the participant to exceed the target wattage for the current phase on three separate and consecutive occasions: <0.5/1.0/1.5/2.0 W/kg body weight for phases 1, 2, 3 and 4, respectively. To reach post-phase 5, participants had to complete six consecutive weeks of training within phase 5.

Each training session consisted of two components. The first component was a supervised aerobic segment conducted on the Total Body Trainer. The duration and structure of this segment were phase-specific: in phases 1 to 4, participants trained continuously for 15 min at a low to moderate intensity (Borg CR10 scale 4–6; 0–10). In phase 5, the duration increased to 17 min and incorporated a 4 min interval segment at a higher but still tolerable intensity (Borg CR10 scale 7–8). Moreover, the training sessions are organized consecutively, allowing each session to build systematically upon its predecessor and thereby ensuring a coherent and progressively structured training process.

The second part of each session comprised approximately 15 min of individualized complementary exercises. These included elements such as breathing techniques, relaxation and coordination training, therapeutic gymnastics, stretching, or low-intensity resistance exercises. The selection and intensity of these elements were tailored to participants’ current functional capacity and adapted as needed in response to symptom fluctuations.

The intervention was designed without a predefined timeframe; therefore, there were no requirements regarding when participants had to complete the program. The progression through the phases was entirely determined by individual symptom trajectories and tolerance. Consequently, the number of participants completing each phase decreased across successive phases. At the time of analysis, only 15 of the 170 enrolled participants had completed phase 5, whereas the severity and fluctuation of symptoms in the remaining participants allowed only slow or partial progression. Importantly, this does not reflect study drop-out but rather the individualized and symptom-responsive nature of the rehabilitation program. The participants who completed all five phases are referred to as “Finishers”. As a result, phase-specific analyses reflect varying sample sizes and should be interpreted as descriptive and exploratory, particularly for later phases.

### 4.5. Sample Size

Table 4 presents the number (*n*) of analyzed samples for each intervention phase. In total, data of *n* = 170 participants were analyzed in this study. For the pre-phase, *n* = 114 samples were analyzed, and for the post-phases, *n* = 100, 60, 34, 19 and 15 were analyzed for post-phases 1 to 5, respectively. A total of *n* = 114 participants started in the pre-phase of the program, while *n* = 34, 16 and 6 participants started in phases 1, 2 and 3, respectively. None of the participants started in phase 4 or 5.

### 4.6. Blood Sampling and Standardization

Venous blood was drawn in the morning under standardized conditions to minimize external influence on hematological and rheological parameters. The participants were in a fasted state and refrained from intense physical activity for 24 h prior to sampling. The blood was collected into either sodium heparin S-Monovette^®^ (Sarstedt AG and Co. KG, Nümbrecht, Germany), BD Vacutainer^®^ 9NC buffered trisodium citrate tubes, or BD Vacutainer^®^ Serum Separating Tubes II Advance Tube (SST) (BD, Becton Dickinson and Company, Franklin Lakes, NJ, USA). The samples containing heparinized blood were processed within one hour of collection, while serum and citrate samples were sent to an accredited clinical laboratory for routine analysis of ferritin, fibrinogen and D-dimer levels.

#### 4.6.1. Complete Blood Count

A complete blood count was determined from whole blood using the Swelab Alfa (Boule Diagnostics AB, Stockholm, Sweden). Only the following RBC-dependent hematological parameters are presented here: red blood cell (RBC) count, hemoglobin concentration (hb), hematocrit (hct), mean corpuscular volume (MCV), mean corpuscular hemoglobin (MCH), mean corpuscular hemoglobin concentration (MCHC), and red blood cell distribution width (RDW).

#### 4.6.2. RBC Rheology

The RBC rheological properties of deformability and aggregation were evaluated using laser-diffraction ektacytometry (LORRCA MaxSis, RR Mechatronics, Zwaag, The Netherlands). Whole blood was centrifuged at 800× *g* for 10 min to separate plasma from the RBC. The plasma supernatant and the RBC pellet were transferred to separate clean tubes, and the intermediate buffy coat layer containing leukocytes and platelets was carefully discarded.

To assess RBC deformability, RBC were suspended in a polyvinylpyrrolidone (PVP, RR Mechatronics) solution with a viscosity of 29.4 centipoise at a dilution ratio of 1:500. The RBC were subjected to controlled shear stresses ranging from 0.3 to 30 Pa using the LORRCA’s Couette system, in which the suspension is sheared between a rotating inner cylinder and a stationary outer cylinder. A laser beam was directed through the suspension, and the diffraction pattern produced by the deforming RBC was captured by a membrane and analyzed by the LORRCA software 5.04 to calculate the elongation index (EI), with the horizontal and vertical axes of the diffraction pattern serving as the basis for the calculation. Two key parameters were derived from the deformability curves: the maximum elongation index (EImax), reflecting the upper limit of RBC deformability, and the shear stress at which half of this maximum was achieved (SS½). The ratio of SS½ to EImax was used as an indicator of membrane deformability, where lower values correspond to a greater deformability of the RBC.

RBC aggregation was assessed by syllectometry based on light transmission changes during aggregation under standardized conditions. For these measurements, RBC were mixed with autologous plasma and adjusted to a final hematocrit of 41%, confirmed via hematology analyzer Swelab Alfa (Boule Diagnostics AB, Spånga, Sweden). The samples were placed on a roller mixer for 15 min to ensure complete oxygenation (Karl Hecht KG, Sondheim vor der Rhön, Germany). The oxygenated samples were introduced into the Couette system, and changes in backscattered light were recorded over 120 s using two photodiodes. The resulting signal was presented as a syllectogram, from which an aggregation index (AI%) was calculated. Additionally, the half-time of aggregation (t1/2) was determined, representing the time required for RBC to reach 50% of their maximal aggregation under the given conditions. The threshold shear rate at which RBC aggregation and disaggregation are balanced was determined through an iterative procedure aimed at calculating dIsc min, the minimum change in backscatter intensity. This parameter corresponds to the lowest shear rate at which RBC aggregates begin to disaggregate (γ at dIsc min, 1/s).

#### 4.6.3. Morphological Analysis

Published protocols were used to determine the morphological abnormalities of RBC. in the finisher subgroup only. Briefly, blood smears were prepared, and the heat was fixed. The smears were stained using the Pappenheim method (Morphisto GmbH, Offenbach am Main, Germany), following the manufacturer’s instructions. This procedure involves the sequential application of May–Grünwald and Giemsa solutions to highlight cellular structures. After staining, the slides were dried and covered using Entellan as a mounting medium. The samples were examined under a Zeiss light microscope coupled with a CCD camera (DXC-1850P, Sony, Berlin, Germany). Morphological features of 200 RBC from ten images were assessed and documented. In this study, the percentage of morphological abnormalities in RBC is reported [15].

### 4.7. Statistical Analysis

The statistical analyses and figure preparation were performed using the statistical software GraphPad Prism (GraphPad software, LLC, Boston, MA, USA; version 9.5.1). The data were first screened for outliers via ROUT (robust regression and outlier removal). The identified outliers were controlled within the dataset and excluded from the analysis if the data could be flagged as unusual compared to previous and subsequent measurements of the same individual. The outliers were not excluded from the analysis if the data appeared consistent or if the values represented personal characteristics such as weight.

Longitudinal changes across phases were analyzed using a linear mixed-effects model (REML) with time as a fixed effect and participant as a random effect to account for within-subject dependence and missing follow-up data. Model assumptions were evaluated by an inspection of residual distributions and QQ-plots, and outcomes were log-transformed where necessary to improve residual normality. If this calculation resulted in a *p*-value below 0.05 (significance level for all statistical tests was set at α < 0.05), a two-stage step-up method of Benjamini, Krieger and Yekutieli (BKY) was applied as a post hoc test to identify phase differences. A comparison of initial values vs. post-phase 5 values of program finishers was tested using either a paired *t*-test or Wilcoxon matched-pairs signed rank test, depending on the normality of the data distribution. The initial values of the total cohort vs. the initial values of finishers were either tested for differences using an unpaired *t*-test or the Mann–Whitney test. Thus, two specific comparisons were performed: (i) initial total vs. initial finishers (unpaired) and (ii) initial vs. post-phase 5 data within the finisher subgroup (paired). No overall three-group comparison was conducted.

Correlation analyses were performed using Spearman’s rank correlation coefficients. In the total cohort, the initial RBC rheological parameters (aggregation index [AI], aggregation half-time [t1/2], and deformability [SS1/2:EImax]) were correlated with hematological indices (MCHC, MCV and RDW) and laboratory markers (Fibrinogen, D-dimers, and Ferritin). In the finisher subgroup, correlations were performed between changes in morphological abnormalities and RBC deformability (Delta (Δ) = post-phase 5 − initial value).

Additionally, to evaluate whether disease duration influenced the baseline values or the magnitude of exercise-induced changes, Spearman’s correlations were calculated between the time since symptom onset and the baseline RBC rheological parameters in the total cohort, as well as between the time since symptom onset and the Δ values in the finisher subgroup. This approach allowed an assessment of whether improvements were likely related to the intervention rather than spontaneous recovery over time. The strength of the Spearman’s correlation coefficient was interpreted as follows: 0–0.3 indicates weak correlation, 0.3–0.5 indicates a moderate correlation, and 0.5–0.7 indicates a strong correlation. Negative values indicate an inverse relationship of corresponding strength.

The data are visualized using violin plots displaying individual values, with the median indicated by a blue line and percentiles shown as black lines. Individual observations are presented as scattered data points to minimize overlap while preserving the underlying data distribution. For figures including pre–post comparisons, inset panels illustrate the individual paired trajectories of finishers between initial and post-phase 5 data.

## 5. Conclusions

In this exploratory longitudinal study, a software-guided, personalized and symptom-responsive exercise intervention was associated with the modulation of RBC rheology in individuals with Long COVID. While conventional hematological, coagulation, and iron metabolism parameters remained stable, the RBC aggregation dynamics were modulated at the cohort level, and program finishers showed distinct adaptations in RBC deformability, accompanied by improvements in RBC morphology. These findings suggest that individualized exercise may stabilize erythrocyte mechanical properties without inducing adverse coagulation responses. However, given the small finisher subgroup and the heterogeneity of Long COVID, the results should be interpreted with caution and require confirmation in larger controlled studies. If confirmed, personalized, software-guided exercise approaches such as the TRIBAL program may represent a scalable and accessible component of comprehensive rehabilitation strategies for Long COVID.

## Figures and Tables

**Table 1:** The hematological values of the analyzed samples per measurement time point (phase). The data are provided as mean (SD).

	Pre	Post-Phase 1	Post-Phase 2	Post-Phase 3	Post-Phase 4	Post-Phase 5	*p*-Value
RBC (×10^6^/µL)	4.7 (0.4)	4.6 (0.4)	4.7 (0.4)	4.8 (0.4)	4.7 (0.4)	4.7 (0.4)	0.3395
Hb (g/dL)	14.2 (1.1)	14.0 (1.1)	14.3 (1.3)	14.7 (1.4)	14.3 (1.4)	14.2 (1.6)	0.1748
Hct (%)	42.1 (3.3)	41.4 (3.2)	42.1 (3.3)	43.1 (3.6)	42.2 (3.7)	41.6 (4.2)	0.2289
MCV (fl)	89.9 (3.3)	90.0 (3.0)	89.8 (3.3)	89.9 (2.8)	89.8 (3.4)	88.9 (3.1)	0.9503
MCH (pg)	30.4 (1.3)	30.6 (1.2)	30.7 (1.4)	30.8 (1.4)	30.5 (1.3)	30.49 (1.3)	0.8013
MCHC (g/dL)	33.7 (0.8)	33.8 (0.8)	34.1 (0.8)	34.1 (0.8)	33.9 (0.8)	34.2 (0.9)	0.0308
RDW (%)	13.3 (0.7)	13.3 (0.8)	13.2 (0.7)	13.2 (0.5)	13.1 (0.6)	13.3 (0.6)	0.8846

**Table 2:** The initial values of the total study cohort (*n* = 170) and the finisher subgroup and post-phase 5 values of the program finisher (*n* = 15). The data are the mean (standard deviation).

	Initial Total	Initial Finisher	Post-Phase 5 Finisher	*p*-Value Initial (Total vs. Finisher)	*p*-Value Finisher (Initial vs. Post-Phase 5)
RBC (×10^6^/µL)	4.7 (0.4)	4.7 (0.3)	4.75 (0.4)	0.4840	0.4237
Hb (g/dL)	14.24 (1.3)	14.1 (1.4)	14.2 (1.7)	0.4079	0.3716
Hct (%)	42.1 (3.3)	41.5 (3.3)	41.6 (4.2)	0.2523	0.4154
MCV (fl)	89.9 (3.2)	88.5 (2.7)	89.1 (3.0)	0.0500	0.0554
MCH (pg)	30.5 (1.4)	30.3(1.2)	30.5 (1.3)	0.3005	0.1940
MCHC (g/dL)	33.8 (0.9)	34.2 (0.8)	34.2 (0.9)	0.0631	0.4458
RDW (%)	13.2 (0.7)	13.0 (0.8)	13.3 (0.6)	0.1336	0.0475

**Table 3:** General information about the study participants at the beginning of the intervention. Data are mean (standard deviation). The *p*-value refers to the difference between the total and finisher subgroups.

	Total	Finisher	*p*-Value
Number, *n*	170 (110 f/60 m)	15 (6 f/9 m)	
Age [years]	49.1 (13.7)	46.7 (14.9)	*p* = 0.1655
Height [cm]	172.3 (9.3)	174.9 (0.7)	*p* = 0.1376
Weight [kg]	80.2 (18.0)	77.4 (12.4)	*p* = 0.3937
BMI ^1^ [kg/m^2^]	27.0 (5.4)	25.3 (3.3)	*p* = 0.1957
PCFS ^2^ [0–4]	2.7 (0.6)	2.2 (0.6)	*p* = 0.0004
PCS ^3^ [0–59]	29.9 (9.6)	26.4 (12.0)	*p* = 0.2898

^1^ BMI = body mass index; ^2^ PCFS = post-COVID-19 Functional Status scale (0: no; 1: negligible; 2: slight; 3: moderate; 4: severe functional limitations); ^3^ PCS = post-COVID syndrome score (≤10.75 = mild; >10.75≤26.5 = moderate; >26.5 = severe).

**Table 4:** Number of analyzed samples per measurement time point and participants in the starting phase of the program.

	Initial	Pre	Post-Phase 1	Post-Phase 2	Post-Phase 3	Post-Phase 4	Post-Phase 5
Number, *n*, of analyzed samples	170 (110 f/60 m)	114 (79 f/35 m)	100 (68 f/32 m)	60 (31 f/29 m)	34 (13 f/21 m)	19 (7 f/12 m)	15 (6 f/9 m)
		Pre	Phase 1	Phase 2	Phase 3	Phase 4	Phase 5
Number, *n*, started in the respective phases		114 (79 f/35 m)	34 (21 f/13 m)	16 (6 f/10 m)	6 (4 f/2 m)	0	0

## Data Availability

The data that support the findings of this study are available upon reasonable request from the corresponding author. The data are not publicly available due to privacy or ethical restrictions.

## Supplementary Materials

The following supporting information can be downloaded at https://www.mdpi.com/article/10.3390/ijms27062671/s1.

## Abbreviations

The following abbreviations are used in this manuscript:

1MSTST	One-minute sit-to-stand test
γ at dIsc min	Aggregation–disaggregation shear rate
AI%	Aggregation index
BMI	Body mass index
Borg-CR scale	Category Ratio Scale, CR10-Scale
CPET	Cardiopulmonary exercise testing
EI	Elongation index
EImax	Maximum elongation index
FACIT	Functional Assessment of Chronic Illness Therapy-Fatigue
Hb	Hemoglobin concentration
Hct	Hematocrit
MCV	Mean corpuscular volume
MCH	Mean corpuscular hemoglobin
MCHC	Mean corpuscular hemoglobin concentration
PCFS	Post-COVID-19 Functional Status scale
PCS	Post-COVID syndrome score
PEM	Post-exertional malaise
PVP	Polyvinylpyrrolidone
RBC	Red blood cell
RDW	Red blood cell distribution width
ROUT	Robust regression and outlier removal
SARS-CoV-2	Severe acute respiratory syndrome coronavirus 2
SS1/2	Shear stress for half-maximal deformation
t1/2	Half-time of aggregation
TRIBAL	Tailored rehabilitation intervention activity for long COVID
VO2_peak_	Peak oxygen uptake
