# Supplementary material for: Personalized Exercise Training Modulates Red Blood Cell Rheology and Morphology in Long COVID
Source: Int J Mol Sci. 2026 Mar 14;27(6):2671. doi: 10.3390/ijms27062671 (PMC13026549; doi:10.3390/ijms27062671)
Supplement: Supplementary file 1 [file ijms-27-02671-s001.zip › ijms-4061548-supplementary.pdf]

Supplementary Table S1. Correlation-based analysis of relationships between the variables. Presented are the r-values and their corresponding p-values (in parentheses).

	**SS1/2:EImax ratio**	**Aggregation Index (%)**	**t1/2 (sec)**	**γ at dIsc min (1/sec)**	**Fibrinogen (mg/dL)**
D-dimer (mg/L FEU)	-0.06036 (p=0.2178)	0.3465 (p<0.001)	-0.3439 (p<0.001)	0.1900 (p=0.0067)	0.2764 (p<0.001)
Fibrinogen (mg/dL)	-0.04373 (p=0.2921)	0.6556 (p<0.001)	-0.6620 (p<0.001)	0.5107 (p<0.001)	
Ferritin (μg/L)	-0.01007 (p=0.4480)	0.03761 (p=0.3127)	-0.006925 (p=0.4642)	0.1693 (p<0.0134)	
MCHC (g/dL)	0.1679 (p=0.0141)				
MCV (fL)	-0.1387 (p=0.0361)				
RDW (%)	0.1361 (p=0.0380)				

R= 0-0.19, Red: very weak – R= 0.2-0.39, Orange: weak – R= 0.4-0.59, Yellow: moderate – R=0.6-0.79, Green: strong relationship. This classification applies to both positive and negative correlations.
